# Correlation between pseudotyped virus and authentic virus neutralisation assays, a systematic review and meta-analysis of the literature

**DOI:** 10.3389/fimmu.2023.1184362

**Published:** 2023-09-18

**Authors:** Diego Cantoni, Craig Wilkie, Emma M. Bentley, Martin Mayora-Neto, Edward Wright, Simon Scott, Surajit Ray, Javier Castillo-Olivares, Jonathan Luke Heeney, Giada Mattiuzzo, Nigel James Temperton

**Affiliations:** ^1^ MRC-University of Glasgow Centre for Virus Research, University of Glasgow, Glasgow, United Kingdom; ^2^ School of Mathematics & Statistics, University of Glasgow, Glasgow, United Kingdom; ^3^ Medicines and Healthcare Products Regulatory Agency, South Mimms, United Kingdom; ^4^ Viral Pseudotype Unit, Medway School of Pharmacy, The Universities of Greenwich and Kent at Medway, Chatham, United Kingdom; ^5^ Viral Pseudotype Unit, School of Life Sciences, University of Sussex, Brighton, United Kingdom; ^6^ Laboratory of Viral Zoonotics, Department of Veterinary Medicine, University of Cambridge, Cambridge University, Cambridge, United Kingdom; ^7^ DIOSynVax, University of Cambridge, Cambridge, United Kingdom

**Keywords:** pseudotype, neutralisation, correlation, virus, live virus correlates of protection

## Abstract

**Background:**

The virus neutralization assay is a principal method to assess the efficacy of antibodies in blocking viral entry. Due to biosafety handling requirements of viruses classified as hazard group 3 or 4, pseudotyped viruses can be used as a safer alternative. However, it is often queried how well the results derived from pseudotyped viruses correlate with authentic virus. This systematic review and meta-analysis was designed to comprehensively evaluate the correlation between the two assays.

**Methods:**

Using PubMed and Google Scholar, reports that incorporated neutralisation assays with both pseudotyped virus, authentic virus, and the application of a mathematical formula to assess the relationship between the results, were selected for review. Our searches identified 67 reports, of which 22 underwent a three-level meta-analysis.

**Results:**

The three-level meta-analysis revealed a high level of correlation between pseudotyped viruses and authentic viruses when used in an neutralisation assay. Reports that were not included in the meta-analysis also showed a high degree of correlation, with the exception of lentiviral-based pseudotyped Ebola viruses.

**Conclusion:**

Pseudotyped viruses identified in this report can be used as a surrogate for authentic virus, though care must be taken in considering which pseudotype core to use when generating new uncharacterised pseudotyped viruses.

## 1 Introduction

Serological assays are an invaluable tool in detecting exposure of pathogens in organisms and understanding the immune system’s response. The level of insight gained from these assays during a disease outbreak is crucial for the initial medical response, and subsequently understanding the dynamics, strength and longevity of the immune response (1–3). An important protective response requires antibody interaction with the pathogen. Upon infection, the humoral response produces antibodies that bind to the antigens displayed by the pathogen, including those that prevent interaction with the receptors necessary for entry into host cells. Assays for antibody analysis have proved effective during recent viral outbreaks, such as those caused by Ebola virus (4, 5) and Severe Acute Respiratory Coronavirus 2 virus (SARS-CoV-2) (6–8), as they allow for detection and monitoring of viral spread in a population. Such assays are similarly applied to animals, which can also identify intermediary hosts or potential reservoirs and provide information about the potential for zoonotic spillover (9, 10), as well as inform on vaccines and treatment efficacy in preclinical studies.

**Figure 1:**
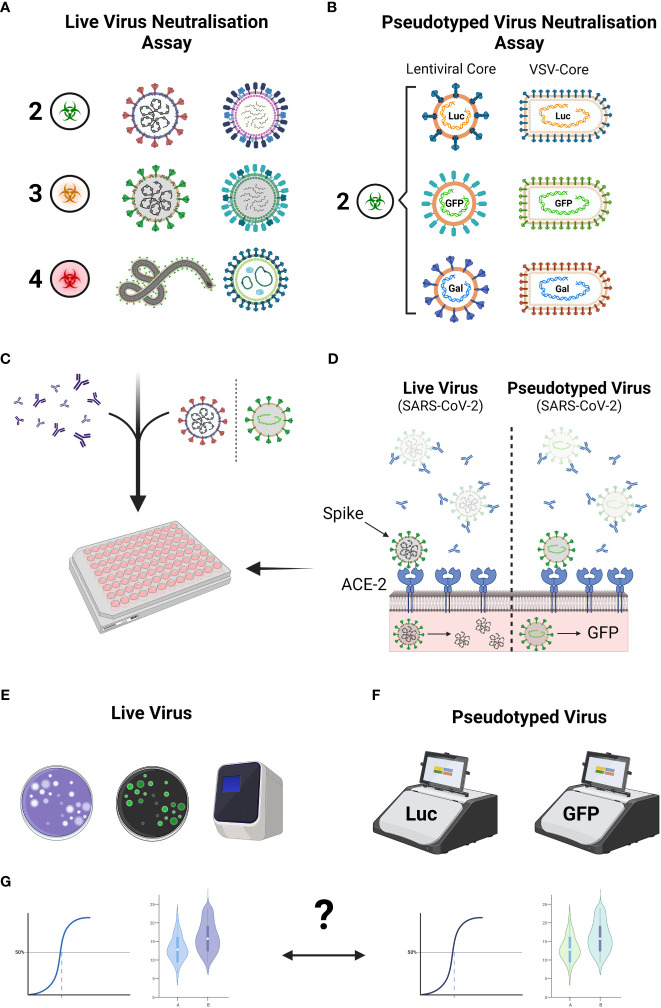
Comparison between live virus neutralisation assay and pseudotyped neutralisation assays. Live viruses are commonly used in neutralisation assays though their practicality may depend on the biohazard containment regulations **(A)**. Pseudotyped viruses, despite displaying glycoproteins of highly pathogenic viruses, are designated as a level 2 pathogen **(B)**. The live virus neutralisation assay and the pseudotyped virus neutralisation assay are designed in a similar fashion whereby antibodies are incubated in the presence of virus, followed by addition of a cell line that is susceptible to virus infection **(C)**. In the context of a SARS-CoV-2 neutralisation assay **(D)**, neutralising antibodies bind to the Spike protein of the virus, preventing the virus to bind to the required entry receptor ACE2. Live viruses that enter begin to replicate, whereas pseudotyped viruses only express the desired reporter gene. Plaque assays, fluorescent staining of viral proteins or qPCR are often used to measure neutralisation levels in live virus assays **(E)**, whereas pseudotyped assays typically rely on measuring the intensities of luciferase or fluorescent protein expression **(F)**. The pertinent question of whether the results derived from either assay correlate still remain **(G)**. Figure created with Biorender.com.

Some serological assays, such as enzyme-linked immuno-absorbance assays (ELISA), can identify the presence of antigen-binding antibodies within a day of receiving a human or animal blood sample (11, 12). When considering antibodies targeting a viral glycoprotein, typically a proportion of the binding antibodies to a viral glycoprotein successfully impair the virus entry, whilst other antibodies bind to non neutralising epitopes, enabling other antibody-mediated immune functions (13). This highlights a shortcoming of binding assays such as ELISAs which lack the functional component of measuring virus entry into cells. Owing to this, in order to measure functional activity, specifically the ability of antibodies in preventing entry, a neutralisation assay is required. These assays are considered the gold standard for measuring the presence and magnitude of neutralising antibodies and typically require the use of authentic virus (14). As a result, these assays often take several days to allow the virus to grow and are subject to biosafety containment requirements depending on the virus under investigation. This restricts the study of viruses classified as hazard group 3 or 4, such as SARS-CoV-2 or Ebola virus and Nipah virus, due to the paucity of facilities that possess such high level of biocontainment. An approach to circumvent these requirements is to use a pseudotyped virus (PV), which can be handled at containment level 2 or below (**Figure 1**). These are comparatively easier to produce, typically by plasmid transfections, and, under optimized conditions, can be produced within 3 to 5 days. Many reviews have been published regarding pseudotype production, core composition, and their uses (15–20). These chimeric viruses commonly use a retroviral or VSV nucleocapsid core are surrounded by a lipid envelope bearing viral glycoproteins of a heterologous virus of interest on their surface. Often, PVs do not contain the virus genomic material required for replication. Instead, the modified genome is replaced by a transgene, for example a reporter gene such as green fluorescent protein (GFP) or luciferase enzyme (16). Upon successful entry into target cells, transgene expression allows for quantification of infected cells. Primarily due to their replication deficiency, PVs can be handled in a containment level 2 laboratories, which are common facilities in biological research laboratories (18). Many viruses of high consequence have been pseudotyped successfully and rapidly during the onset of an outbreak, as authentic viruses typically require isolation and stock amplification, whereas PVs require a published sequence of the viral glycoprotein to be cloned into an expression plasmid. Due to their external mimicry of the virus of interest, with reduced risk of acquiring mutations during production in mammalian tissue culture as seen with authentic viruses, PVs are an effective tool to use in neutralisation assays (18, 19). The COVID-19 global pandemic, caused by SARS-CoV-2, caused a significant rise in the use of pseudotype assays for both serology and molecular virology studies (17, 21). When PVs are used in a multi-well plate assay setting they are often referred to as pseudotype virus microneutralisation assays (pMNA). For the purposes of this systematic review, the alternative authentic virus microneutralisation assay will be referred to as vMNA.

Given that neutralising antibodies are one of the principle components measured to determine correlates or surrogates of protection against disease or infection (22–24), the neutralisation test remains a critical assay. An important aspect when determining a correlate or surrogate of protection is to be able to draw comparisons between data and bridge between studies. By calibrating assays to a common reference reagent, often a pooled sera sample, assay readouts can be standardised across laboratories worldwide as these relative results are reported in a standard unitage (25–27). It is important that such common reagent is used correctly to calibrate in house standards, but in some cases, this is still not enough and the reduction of inter-laboratory variation can only be achieved by sharing common protocols and critical reagents similar to the approach used by the CEPI Centralised Laboratories network. Such reference reagents have been produced for several viruses, including many of high consequence which are applicable to pseudotyping (28–30). Whilst reporting results relative to a reference reagent reduces inter-laboratory variations and allows comparisons between assays, it is fundamentally important to investigate whether surrogate assays, designed to mimic and replace vMNAs which employ highly pathogenic viruses, correlate. If there is a correlation between a pMNA and a vMNA, then the results from either assay could be applied within clinical trials and investigations aimed at identifying the correlates for protection against a virus.

However, it is commonly queried how well the results from a pMNA correlate with those from a vMNA. The question is particularly relevant with the increasing uptake of pMNAs as a consequence of the recent COVID-19 pandemic and their increasing application to clinical trials as focus turns to vaccine development for other high consequence pathogens (31, 32). The studies to-date use a mixture of correlation formulae, most of which are Pearson’s R and/or Spearman’s Rho (33, 34). Other studies have instead fitted linear regressions to understand the relationship between the two variables, with the R^2^ value providing an equivalent measure to the square of Pearson’s R in the case of a positive relationship (35). Several reviews on PVs or neutralisation assays have included some of these studies which sought to correlate results from both assays, yet only a handful are cited (17–19). Despite several studies directly comparing PV and authentic virus neutralization assays, correlation information tends to be buried in the mass of data or supplementary material in these reports. It is likely that for these reasons, the question as to whether the two assays correlate is still frequently posed.

To the best of our knowledge, there is no systematic review nor meta-analysis that has condensed the literature that has correlated pMNA and vMNA. Therefore, the purpose of this systematic review and meta-analysis is to collect the available information on the comparison between the two tests, analyse the strength of correlations, and present the results in a clear and coherent manner. Overall, we aim to inform the wider community whether pseudotyped viruses can be used as surrogates for authentic virus for the purposes of a neutralisation assay and subsequently to determine the correlates of protection against a virus. Despite the findings within this report, it remains critical that PV-based assays continue to be assayed and correlated with authentic virus wherever possible, particularly if a new PV has been designed for use. Given that correlation coefficient values have different classifications of strength based on the field of study, we included a table based on the definitions that are often cited in the field of medicine (34, 36, 37) (**Table 1**).

**Table 1:** Guide for interpreting correlation coefficients in the medical field of study.

Correlation Coefficient value	Strength of Relationship
>0.8	Very strong
0.6 - 0.79	Moderately strong
0.3 - 0.59	Fair
<0.3	Poor

## 2 Methods

### 2.1 Search strategy and selection criteria

Google Scholar and PubMed were used to identify published research articles which reported data on correlation between pMNA and vMNAs. The following Boolean search terms were employed to filter studies indexed in Google Scholar and PubMed: “pseudotype|pseudotyped|pseudoparticle” “correlate|correlated|correlation” “live” “virus” “neutralisation|neutralization”.

The criteria for inclusion were reports that contained neutralisation assays with both pseudotype virus and authentic virus, as well as application of a mathematical formula to assess the relationship between the results, either by linear regression, Pearson’s correlation, Spearman’s rank, or a combination of the three. Studies that did not present any form of analysis of correlation were excluded.

### 2.2 Data collection

We extracted the following data from reports that satisfied our selection criteria: report author name and year, virus used, pseudotype core used, neutralisation assay readout (both for pMNA and vMNA), correlation method, p value of the correlation coefficients, number of samples, and sample types. In total, we identified 67 reports that satisfied our selection criteria and were used for comparative data analysis.

### 2.3 Statistical analysis

For our meta-analysis, we considered data for the relationships between SARS-CoV-2 PVs and authentic virus. There was insufficient data to consider other viruses in separate meta-analyses and we decided not to analyse the results from multiple viruses together. We instead present the data for other viruses in a table in the supplementary materials (**Suppl. Table 1**). For the studies reporting a linear regression (R^2^), we opted to convert the value by its square-root, so that it may be combined with the Pearson’s R values derived from other studies and therefore included in the analysis. We checked that all regressions reported only included the PVs and authentic virus and that the relationships were all positive. We did not have sufficient Spearman’s Rho values to analyse and these cannot be directly combined with the Pearson’s R values, as they do not measure the same characteristic. Therefore, we did not attempt to carry out a meta-analysis of Spearman’s Rho coefficients. These values are reported in the supplementary materials (**Suppl. Table 1**). We therefore used a dataset of 50 Pearson’s R coefficients from 22 papers. Since studies on SARS-CoV-2 used different PV cores (HIV and VSV), PV assays (eGFP, GFP, Luciferase, PRNT and SEAP) and sample types (hamster sera, human mAbs, human plasma and human sera), we checked for differences in the Pearson’s correlations between studies using t-tests with a null hypothesis of no difference in the mean Pearson’s correlations between the groups containing at least 10 results (**Suppl. Figure 1**). Since we failed to reject the null hypothesis for any comparison, we decided to carry out our meta-analysis on the full dataset. We had only very limited results reported for different SARS-CoV-2 variants, so that investigating differences in results for each variant alone is left for future work. The analysed datasets used identical variants for PV and authentic viruses.

We conducted a three-level meta-analysis of Fisher’s z-transformed Pearson’s correlations, using the inverse-variance method, accounting for the dependence between multiple results from the same study (38, 39). We assigned data to “clusters” based on their dependence on other data. All coefficients calculated using the same dataset were considered dependent and were assigned to the same cluster, resulting in 26 clusters in total. Taking the example of Wang et al, 2020 (40), a correlation coefficient was calculated for each of two independent datasets, so that these two coefficients were assigned to separate clusters, while Sholukh et al, 2021 (41) presented four correlation coefficients that were calculated using the same datasets, so that these coefficients were all assigned to the same cluster. Clusters with higher estimated sampling variance of their correlation coefficients, e.g., due to lower sample sizes, are given lower weights in the calculation of the pooled correlation, while clusters are given higher weights if there is less dependence among their correlation coefficients (39). The heterogeneity variance, τ^2^, was calculated using the restricted maximum likelihood estimator, with confidence interval estimates calculated using the profile likelihood method. We assessed heterogeneity using the I^2^ and H statistics (42) and we calculated prediction intervals (using the t-distribution) for the pooled correlation estimate. While confidence intervals provide measures of uncertainty around the true mean values of correlation, the prediction interval provides a measure of uncertainty around the likely values of correlation to be seen in future studies (38). We checked for influential outliers by removing correlations in turn and recalculating all estimates. We plotted Fisher’s z-transformed correlation against standard error (a “funnel plot”) to assess possible publication bias. All calculations were carried out in R version 4.3.1 (R Core Team, 2022) using the packages meta (43), metafor (44) and dmetar (45).

## 3 Results

### 3.1 Results of literature search

Our search terms returned a total of 33 reports in PubMed and 5,880 reports in Google Scholar. After manually screening abstracts and titles, we identified 80 studies that met our selection criteria and ultimately included 67 reports in this systematic review (**Suppl. Table 1**). The primary reason for exclusion were reports that either did not include both pMNA and vMNA, or reported neutralisation titres in both the pMNA and vMNA, but did not carry out a correlation analysis between the two methods. Briefly, the total number of reports found for each virus were; SARS-CoV-2 (n=32) (40, 41, 46–75), SARS-CoV-1 (n=2) (76, 77), Canine distemper virus (CDV, n=1) (78), Chikungunya virus (CHIKV, n=1) (79), European bat lyssavirus 1 (EBLV-1, n=1) (80), EBLV-2 (n=1) (80), Ebola virus (EBOV, n=3) (81–83), Hepatitis C virus (HCV, n=3) (84–86), Human immunodeficiency virus (HIV, n=1) (87), Hantaan orthohantavirus (HTNV, n=2) (88, 89), Influenza A virus H5N1 (IAV H5N1, n=5) (90–94), IAV H7N9 (n=1) (95), Japanese encephalitis virus (JEV, n=1) (96), Lagos bat virus (LBV, n=1) (97), Middle East respiratory syndrome virus (MERS, n=4) (98–101), Newcastle disease virus (NDV, n=1) (102), Nipah virus (NIV, n=1) (103), Peste des petite ruminants virus (PPRV, n=1) (104), Puumala virus (PUUV, n=1) (105), Rift Valley fever virus (RVF, n=1) (106), Rabies virus (RABV, n=2) (107, 108), and Seoul orthohantavirus (SEOV, n=2) (88, 89). A summary of the findings from these reports can be viewed in **Table 2**, whereas a more detailed breakdown for each report can be viewed in the supplementary file (**Suppl. Table 1**).

**Table 2:** Summary of reported correlation coefficients. The bounds represent the minimum and maximum point values across the studies.

Virus	No. of Reports	Correlation Range (Linear R^2^)	Correlation Range (Pearson’s)	Correlation Range (Spearman’s)	Correlation Range (Intra-Class)
Severe acute respiratory syndrome coronavirus 2 (SARS-CoV-2)	31	0.385 - 0.993	0.641 - 0.939	0.54 - 1	0.872 - 0.872
Severe acute respiratory syndrome coronavirus 1 (SARS-CoV-1)	2	–	0.69 - 0.78	–	–
Canine distemper Virus (CDV)	1	–	–	0.65 - 0.91	–
Chikungunya virus (CHIKV)	1	0.78 - 0.98		–	–
European bat 1 lyssavirus (EBLV-1)	1	–	0.79 - 0.79	–	–
European bat 2 lyssavirus (EBLV-2)	1	–	0.9 - 0.9	–	–
Ebola virus (EBOV)	3	–	0.96 - 0.96	0.54 - 0.86	–
Hepatitis C virus (HCV)	3	–	0.893 - 0.893	0.7 - 0.93	–
Human immunodeficiency virus (HIV)	1	0.903 - 0.903	–	–	–
Hantaan virus (HTNV)	1	0.91 - 0.91	–	–	–
Influenza A virus H5N1 (IAV H5N1)	5	0.524 - 0980	0.734 - 0.78	0.79 - 0.79	–
Influenza A virus H7N9 (IAV H7N9)	1	–	0.82 - 0.82	–	–
Japanese encephalitis virus (JEV)	1	0.915 - 0.915	–	–	–
Lagos bat lyssavirus (LBV)	1	–	0.83 - 0.83	–	–
Middle East respiratory syndrome virus (MERS)	4	0.96 - 0.96	0.88 - 0.934	0.97 - 0.97	–
Newcastle disease virus (NDV)	1	0.92 - 0.92	–	–	–
Nipah virus (NIV)	1	–	–	–	–
Peste des petits ruminants virus (PPRV)	1	–	–	0.89 - 0.89	–
Puumala virus (PUUV)	1	–	–	0.82 - 0.82	–
Rift Valley fever virus (RVF)	1	–	–	0.77 - 0.77	–
Rabies virus (RABV)	3	0.946 - 0.946	0.915 - 0.918	–	–
Seoul orthohantavirus (SEOV)	1	0.82 - 0.845	–	–	–

Some types of correlation coefficient were not reported in any studies of some viruses and this is indicated by entries containing only "-".

Aside from SARS-CoV-2 which will be analysed in the following sections of this study, we found that in general, most of the pseudotypes correlated well with the vMNA, irrespective of pseudotype cores and the readout techniques used to measure the assay results (**Suppl. Table 1**.). We found some studies that did not clarify the correlation test used, and were therefore omitted from **Table 2**, though relevant information including the r value is still included in the **Supplementary Table 1**. Interestingly, a study analysing the EBOV PVs reported that the choice of the PV core had a substantial impact on correlation with authentic virus (82, 83). When the negative control samples were omitted from the neutralisation assays, the correlation coefficients dropped from 0.68, 0.77 to -0.03 and 0.18, effectively showing no correlation, whereas the samples assayed with the VSV core PVs retained correlation coefficients of 0.84 and 0.96 (**Suppl. Table 1**.). This study highlights the need to consistently verify whether cores of pseudotypes can affect correlations with vMNAs.

### 3.2 Three-level meta-analysis results

**Figure 2:**
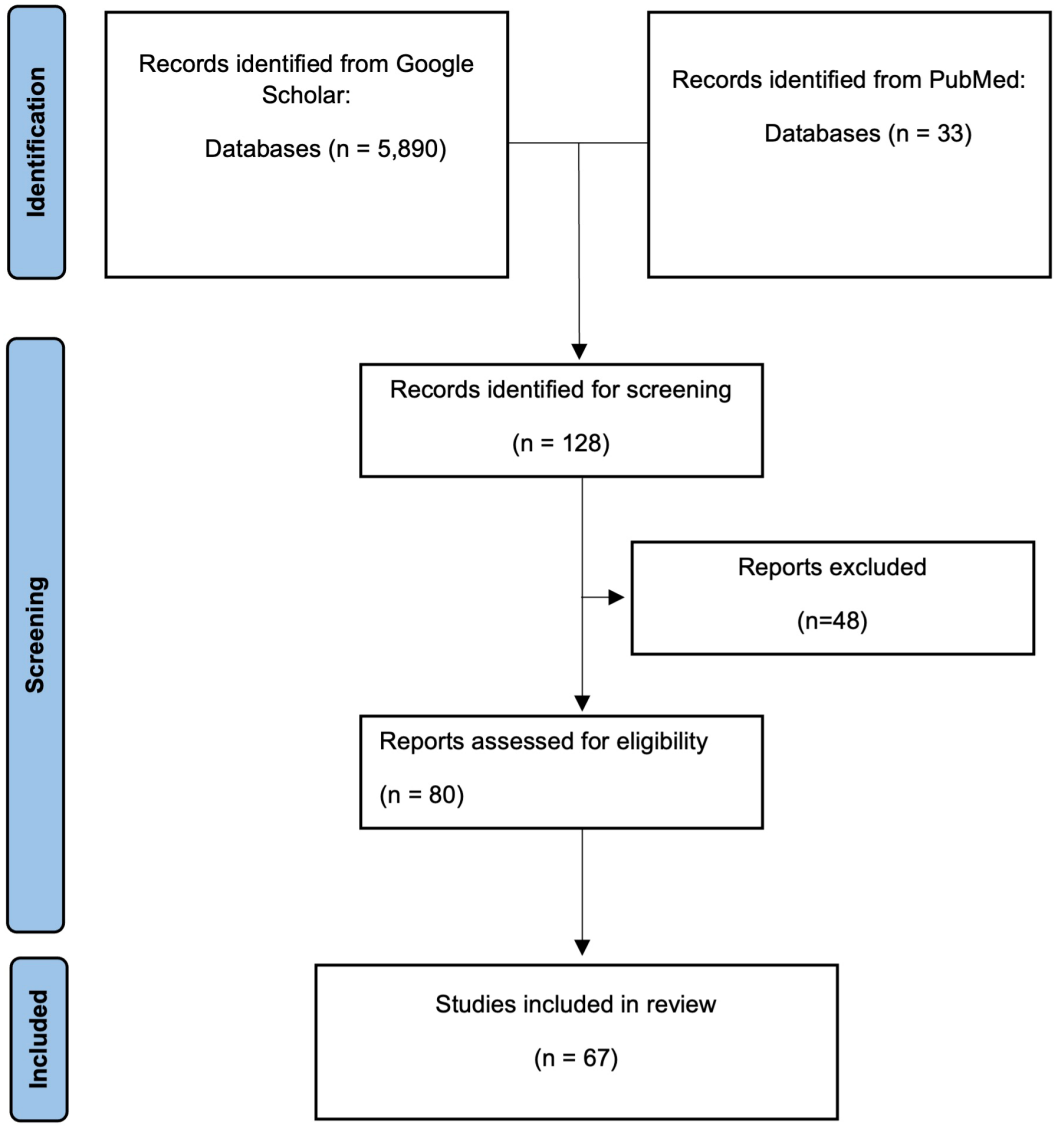
Flow diagram of the study identification and selection process.

From 22 SARS-CoV-2 studies we analysed 50 Pearson’s correlation coefficients, which were derived from a combined total of 1238 data points by pMNA and vMNA (**Figure 2**). As stated in the methods, we verified that there were no significant differences in the mean Pearson’s correlation values between studies that used different PV cores, neutralising reagents and assay readout types (**Suppl. Figure 1**). We calculated a pooled correlation of 0.86 (95% CI; 0.82-0.89, p < 0.01). These results suggest that there is a strong correlation between the results derived by pMNA and vMNA.

The results indicated the presence of low to moderate between-cluster heterogeneity [I^2 ^= 37.1% (CI: 11.2%-55.5%); H=1.26 (CI: 1.06 to 1.50); τ^2 ^= 0.05 (CI: 0.02-0.12)]. This means that there is some weak evidence of differences in the true effect sizes in the study. A 95% prediction interval (PI) for the pooled correlation is 0.69-0.94, which means that it is highly likely that the true correlation between pMNA and vMNA in a future study will lie between 0.69 and 0.94. Since this is entirely greater than 0.5, this provides us with evidence of a positive relationship between pMNA and vMNA for SARS-CoV-2, appropriately accounting for the distribution of effects amongst the studies. Removing results in turn did not lead to substantial reductions in heterogeneity. Our “funnel plot” (**Suppl. Figure 2**) shows that most points lie within the funnel shape in a symmetrical pattern, providing no evidence of publication bias.

**Figure 3:**
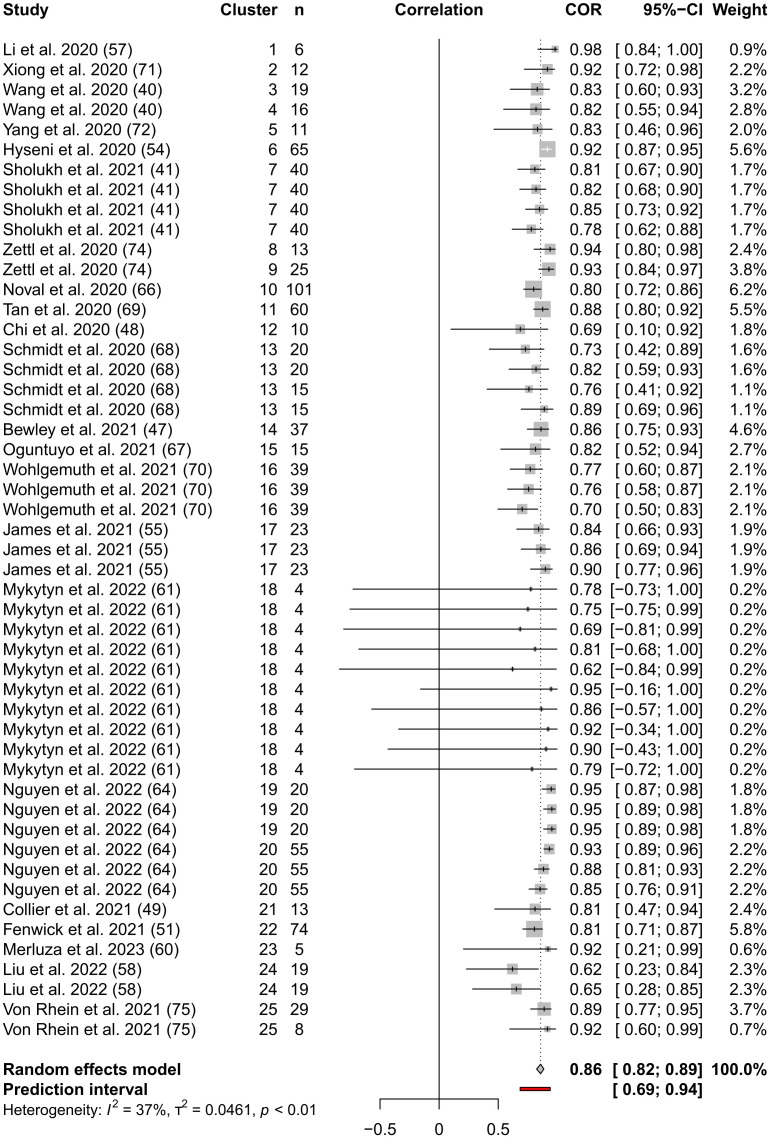
Forest Plot of the three-level meta-analysis results. The endpoints of the black or white horizontal lines represent the endpoints of the 95% CIs for the Pearson’s correlation coefficients for each study. The grey boxes represent the sample sizes of each study. The vertical dotted line represents the pooled Pearson’s correlation coefficient estimate and the grey diamond represents the 95% CI for the pooled Pearson’s correlation coefficient estimate. The 95% prediction interval is shown by the red line. The table columns are, respectively, study name, cluster indicator, sample size (n) from which Pearson’s correlation coefficient was calculated, correlation as described above, Pearson’s correlation coefficients, 95% CI of Pearson’s correlation coefficients, and weighting assigned to each coefficient.

Our “forest plot” (**Figure 3**) shows the calculated interval estimates for each study. We note that the majority of the interval estimates include our pooled estimate and that all studies except Mykytyn et al. (61), which has very small reported sample sizes, have entirely positive interval estimates.

### 3.3 Agreement between pMNA and vMNA by Bland-Altman method

Since Pearson’s or Spearman’s correlation coefficients are used for understanding correlation between two variables, they may not determine whether different assays are strictly in agreement with each other. The Bland-Altman method (109) is a frequently applied analysis which is often used to determine agreement between two methods that aim to measure the same variable, in this case, antibody neutralising capability. Within our literature search, several studies have used the Bland-Altman method of analysis. Therefore, we also refined the literature search used for this study by adding the search terms; “Bland-Altman”. All four resulting papers identified were already included from the main literature search. Due to the power of this statistical method, we opted to present the results by the Bland-Altman method within the reports in a separate table (**Table 3**). All studies that reported results from the Bland-Altman method showed high levels of agreement between pMNA and vMNA.

**Table 3:** Reported Bland-Altmann results.

Study	Virus	Samples	Conclusions
Hyseni *et al.*, 2020 (54)	SARS-CoV-2	65	64/65 samples within 95% Limit of Agreement
Lester *et al.*, 2019 (100)	MERS	52	High level of agreement
Nie *et al.*, 2017 (107)	RABV	320	All samples within Limit of Agreement
Buchy *et al.*, 2010 (91)	IAV H5N1	41	High level of agreement

## 4 Discussion

Given the interest in the results derived by pMNA compared to vMNA, our systematic review and meta-analysis sought to consolidate the data to inform the wider community on whether there is a correlation and subsequently, agreement between the two assays. The results of the meta-analysis would confirm that for SARS-CoV-2 there is a strong degree of correlation between pMNA and vMNA. Despite the limited number of studies, the Bland-Altman results presented in this manuscript also indicate a high level of agreement between the two assays. This data support the use of pMNA as a surrogate to the vMNA, though more correlation studies by Bland-Altman would be very valuable to perform in future reports.

Moreover, since multiple viral cores can be used for pseudotyping, it is important to assess whether this could impact the pMNA vs vMNA correlation. It would appear that in the case of the Ebola virus, there is a lower concordance, if a lentiviral core is used in the pMNA compared with a VSV core (82, 83). Whilst the precise reason for influence of the core remains unknown, though speculated to be due to the morphological difference between a VSV capsid and a filamentous EBOV particle (82) or the target cells, which is the same for the authentic virus and EBOV-VSV but differ for the lenti-based pMNA. It will be important to determine whether these differences exist in the case of other filoviruses and indeed other viruses, as there may be a high risk of reporting erroneous results. Therefore, it is important to optimize all aspects of the pMNA and different pseudotype cores combined with identical envelope glycoproteins should always be assessed in parallel with the authentic virus in neutralization tests, if possible. Critically, the two EBOV studies observed the reduced correlation of the lentiviral cores when negative control sera were excluded from their analyses. Therefore, we advise future correlation studies to consider not only including negative control samples within their analyses, but also consider deriving correlations with and without the negative control samples, especially if the number of samples is low and multiple cores are under assessment.

Interestingly, multiple studies have mentioned that one of the benefits of using PVs is that they are more sensitive in discriminating samples containing weaker or a low concentration of neutralising antibodies (92, 100, 104). In fact, one report provided evidence of the vMN assay reporting false negative results on samples that contained neutralising antibodies, successfully detected by the pMN (102). Whilst this would highlight the benefits of using PVs for detecting positive samples within a human or animal population, it is may also bring into question whether the results derived from the weaker samples could protect the individual or animal from subsequent infection, given that the authentic virus was not neutralised. However, it is essential to consider that lower limits of detections can change based on assay design, virus species, the titre of the virus used, and the volume of serum sample used. This highlights reporting of results relative to a reference reagent can add value by enabling comparisons between data produced by different methods. Whilst use of a reference material will not ultimately improve assay performance, it helps to highlight differences. In any case, having a more sensitive assay such as the pMNA would prove to be very useful for epidemiological studies that are aiming to determine whether a virus exists or existed in a particular human or animal population, as opposed to correlating neutralising titres towards disease severity or protection.

Lastly, it is very important to distinguish the type of interpretation derived from either Pearson’s R or Spearman’s rank correlation analyses and the Bland-Altman plot. Neither the Pearson’s R, which is a measure of the linear relationship between two variables, nor the Spearman’s rank, that informs on correlation from measurements taken on an ordinal scale, provide information on the agreement between two different assays. In this case, the Bland-Altman method is required (109). Our literature search has shown for multiple viruses that the pMNA and vMNA have high agreement for multiple viruses in several families.

The main limitation of our systematic review is that it was biased towards SARS-CoV-2, due to the sheer number of publications dedicated to this virus in the past three years, providing enough correlation values that allowed for the meta-analysis. Whilst it would have been useful to carry out the same analysis for other viruses, unfortunately there were not enough correlation values. We did not use the Spearman’s Rho coefficients in our analyses, but the strong positive values of these, for both SARS-CoV-2 and other viruses (**Suppl. Table 1**) do not disagree with our main conclusions that PVs and authentic virus showed strong positive relationships. Some of the studies used very small sample sizes, which was accounted for through giving lower weights to these studies. We opted to include studies that used PVs that are non-replicative, single cycle of infection, therefore excluding studies that used replicon infection systems, despite some of these reports showing high correlation and high level of agreement between single-round replicons and authentic virus in a neutralisation assay (110, 111). Lastly, new virus and cell-free assays have now been developed for SARS-CoV-2 that measure the capability of antibodies blocking the spike protein from interacting with its receptor ACE-2, effectively becoming a surrogate neutralisation assay, have shown to have strong correlations with both pMNAs and vMNAs (51, 69, 112–114). Whilst these assays do not fit the scope of this study, we believe it is worth mentioning and monitoring for follow up meta-analyses.

In summary, our systematic review and meta-analysis shows that the pMNA designed for use towards SARS-CoV-2 serological studies demonstrated a high degree of correlation with assays performed using the authentic virus. In addition, many other viruses that have been pseudotyped also show a high degree of correlation. We recommend, where possible, that future studies on methods agreement should continue to investigate the use of multiple PV cores, to determine whether there could be differences in neutralisation titres, such as that exemplified with Ebola virus PVs. It is also essential that future studies incorporate the Bland-Altman analysis to determine the agreement between the two assays as well as this is substantially more informative, especially when both assay results are to be applied to clinical trials and assessed for determining correlates of protection. Ultimately, we would encourage laboratories to calibrate assays to reference materials, if one is available and relevant for the isolate under study, which will support these future comparisons and critically provide traceability to a correlate of protection once derived.

## Data availability statement

The original contributions presented in the study are included in the article/**Supplementary Material**. Further inquiries can be directed to the corresponding author.

## Author contributions

DC and NT conceptualised the study. DC, CW, EB, MM-N, EW, SS, SR, JC-O, JH, GM assisted in the literature search and proof-reading of the manuscript. CW and SR carried out the statistical analysis. GM, JC-O, JH and NT provided critical evaluation of the manuscript. All authors contributed to the article and approved the submitted version.
