# Supplementary material for: Correlation between pseudotyped virus and authentic virus neutralisation assays, a systematic review and meta-analysis of the literature
Source: Front Immunol. 2023 Sep 18;14:1184362. doi: 10.3389/fimmu.2023.1184362 (PMC10544934; doi:10.3389/fimmu.2023.1184362)
Supplement: Supplementary file 1 [file Image_1.pdf]

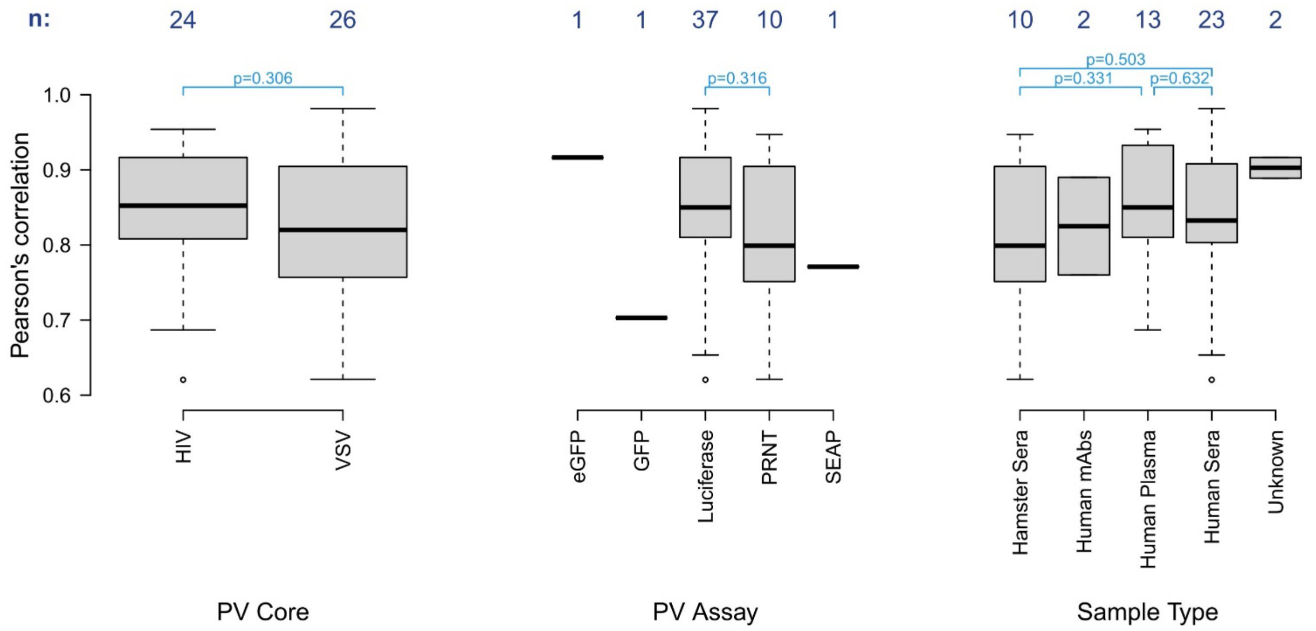

Supplementary Figure 1. Boxplots of Pearson's correlation coefficients for: studies with HIV and VSV PV Cores (left); eGFP, GFP, Luciferase, PRNT and SEAP PV Assays (centre); and hamster sera, human mAbs, human plasma, human sera and unknown/unspecified Sample Type (right). P-values for t-tests of differences in means between groups are shown in light blue, with horizontal light blue lines linking the groups being compared. The numbers of studies in each group are shown in dark blue.
