# Supplementary material for: Correlation between pseudotyped virus and authentic virus neutralisation assays, a systematic review and meta-analysis of the literature
Source: Front Immunol. 2023 Sep 18;14:1184362. doi: 10.3389/fimmu.2023.1184362 (PMC10544934; doi:10.3389/fimmu.2023.1184362)
Supplement: Supplementary file 2 [file Image_2.pdf]

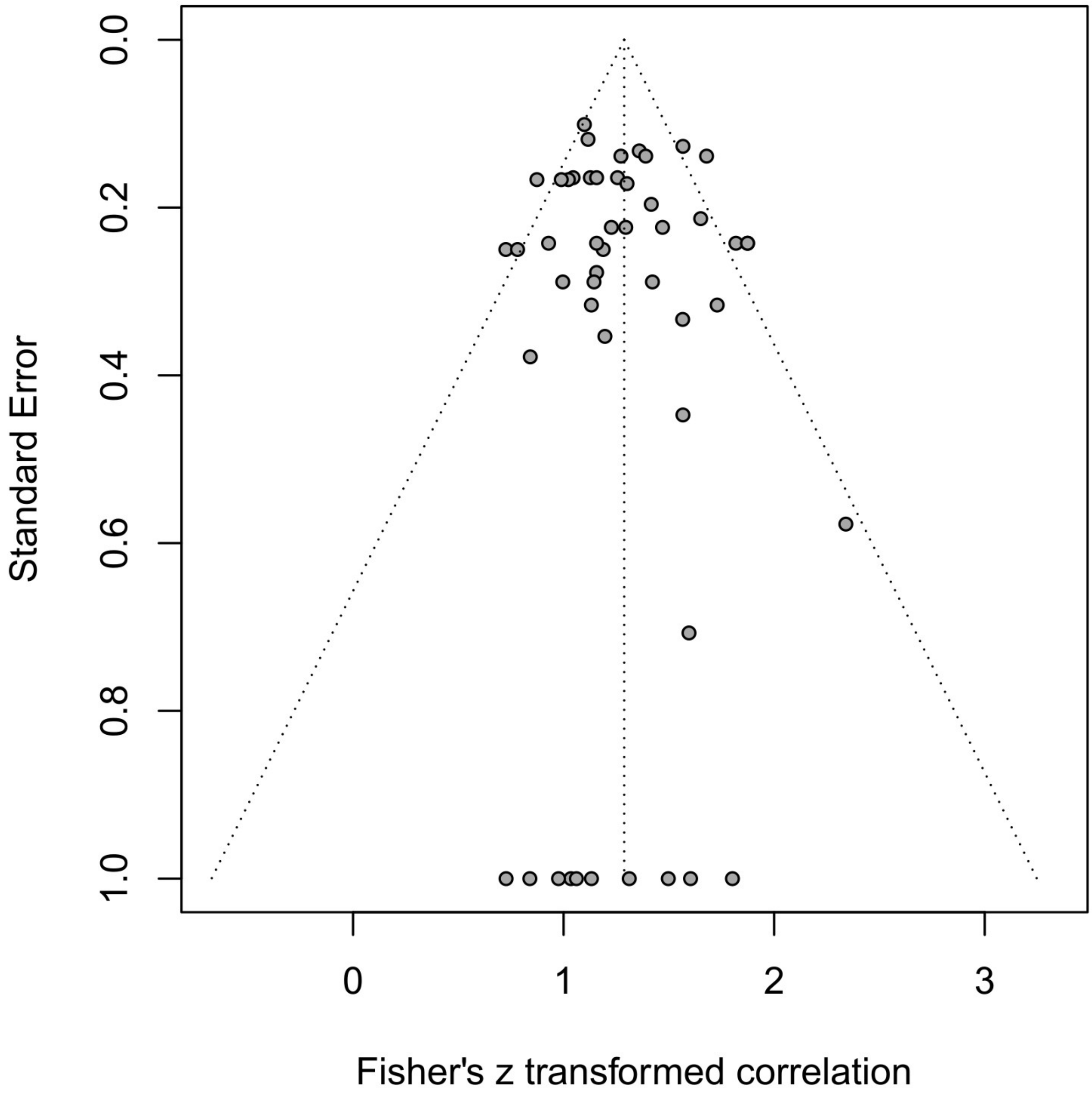

Supplementary Figure 2. “Funnel plot”, showing Fisher’s z-transformed correlation against standard error for each reported correlation. The dotted lines represent the theoretical shape within which 95% of points are expected to lie when no biases or heterogeneity are present.
