# Supplementary material for: Correlation between pseudotyped virus and authentic virus neutralisation assays, a systematic review and meta-analysis of the literature
Source: Front Immunol. 2023 Sep 18;14:1184362. doi: 10.3389/fimmu.2023.1184362 (PMC10544934; doi:10.3389/fimmu.2023.1184362)
Supplement: Supplementary file 3 [file Table_1.docx]

| **Paper**  **Supplementary Table 1**. List of reported correlation coefficients. *denotes result with inclusion of a chosen pre-pandemic, or pre-vaccinated, or non-neutralising control sample within the correlation analysis, as described within the cited manuscript. | **Virus** | **PV Core** | **LV Assay** | **PV Assay** | **Correlation** | **Method** | **Sample size** | **Sample type** |
| --- | --- | --- | --- | --- | --- | --- | --- | --- |
| Atti *et al*. 2022 (46) | SARS-CoV-2 | HIV | Fluorescence | Luciferase | 0.78 (IC50) (p<0.0001) | Spearman's | 92 | Human sera |
|  |  |  |  |  | 0.63 (IC50) (p<0.0031) | Spearman's | 23 | Human sera |
|  |  |  |  |  | 0.61 (IC50) (p<0.0001) | Spearman's | 92 | Human sera |
|  |  |  |  |  | 0.54 (IC50) (p=0.014) | Spearman's | 23 | Human sera |
| Bewley *et al*. 2021 (47) | SARS-CoV-2 | VSV | PRNT | Luciferase | 0.862 (IC50) (p<0.001) | Pearson's | 37 | Human sera |
| Chi *et al*. 2020 (48) | SARS-CoV-2 | HIV | qPCR | Luciferase | 0.6868 (IC50) (p=0.0283) | Pearson's | 10 | Human plasma |
| Collier *et al*. 2021 (49) | SARS-CoV-2 | HIV | Coomassie Intensity | Luciferase | 0.8117 (IC50) (p<0.001) | Pearson's | 13 | Human sera |
| D'Apice *et al*. 2022 (50) | SARS-CoV-2 | HIV | CPE | Luciferase | 0.9231* (IC90) (p<0.0001) | Spearman's | 111 | Human sera |
|  |  |  |  |  | 0.9139 (IC90) (p<0.0001) | Spearman's | 29 | Human sera |
|  |  |  |  |  | 0.8444 (IC90) (p<0.0001) | Spearman's | 59 | Human sera |
|  |  |  |  |  | 0.9486 (IC90) (p<0.0001) | Spearman's | 29 | Human sera |
|  |  |  |  |  | 0.8844 (IC90) (p<0.0001) | Spearman's | 59 | Human sera |
|  |  |  |  |  | 0.7341 (IC90) (p<0.0001) | Spearman's | 29 | Human sera |
|  |  |  |  |  | 0.5376 (IC90) (p<0.0001) | Spearman's | 59 | Human sera |
| Fenwick *et al*. 2021 (51) | SARS-CoV-2 | HIV | CPE | Luciferase | 0.65 (IC50) | Linear R^2^ | 74 | Human sera |
| Grzelak *et al*. 2020 (52) | SARS-CoV-2 | HIV | CPE | GFP | 0.83 (Single Dilution) (p<0.0001) | Spearman's | 21 | Human sera |
| Harvala *et al*. 2020 (53) | SARS-CoV-2 | HIV | ELISA | Luciferase | 0.83 (IC50) (p<0.0001) | Spearman's | 51 | Human plasma |
| Hyseni *et al*. 2020 (54) | SARS-CoV-2 | HIV | CPE | Luciferase | 0.84 (IC50) (p<0.0001) | Linear R^2^ | 65 | Human sera |
|  |  |  |  |  | 0.872 (IC50) (p<0.0001) | Intra-Class | 65 | Human sera |
| James *et al*. 2021 (55) | SARS-CoV-2 | HIV | CPE | Luciferase | 0.7084 (IC50) | Linear R^2^ | 23 | Human sera |
|  |  |  |  |  | 0.7405 (IC50) | Linear R^2^ | 23 | Human sera |
|  |  |  |  |  | 0.8092 (IC50) | Linear R^2^ | 23 | Human sera |
| Legros *et al*. 2020 (56) | SARS-CoV-2 | MLV | CPE | GFP | 0.75* (IC50) (p<0.0001) | Spearman's | 140 | Human sera |
| Li *et al*. 2020 (57) | SARS-CoV-2 | VSV | PRNT | Luciferase | 0.9636 (IC50) (p<0.001) | Linear R^2^ | 6 | Human sera |
| Liu *et al*. 2022 (58) | SARS-CoV-2 | HIV | PRNT | Luciferase | 0.385 (IC50) | Linear R^2^ | 19 | Human sera |
|  |  | VSV | PRNT | Luciferase | 0.427 (IC50) | Linear R^2^ | 19 | Human sera |
| Maciola *et al*. 2022 (59) | SARS-CoV-2 | VSV | OD (Crystal Violet) | Luciferase | 0.78 (IC50) (p<0.0001) | Spearman's | 60 | Human sera |
| Merluza *et al*. 2023 (60) | SARS-CoV-2 | HIV | PRNT | Luciferase | 0.921 (IC50) (p=0.0263) | Pearson's | 5 | Human sera |
| Mykytyn *et al*. 2022 (61) | SARS-CoV-2 | VSV | PRNT | PRNT | 0.6018 (IC50) | Linear R^2^ | 4 | Hamster sera |
|  |  |  |  |  | 0.5645 (IC50) | Linear R^2^ | 4 | Hamster sera |
|  |  |  |  |  | 0.4701 (IC50) | Linear R^2^ | 4 | Hamster sera |
|  |  |  |  |  | 0.659 (IC50) | Linear R^2^ | 4 | Hamster sera |
|  |  |  |  |  | 0.3858 (IC50) | Linear R^2^ | 4 | Hamster sera |
|  |  |  |  |  | 0.8968 (IC50) | Linear R^2^ | 4 | Hamster sera |
|  |  |  |  |  | 0.7478 (IC50) | Linear R^2^ | 4 | Hamster sera |
|  |  |  |  |  | 0.8503 (IC50) | Linear R^2^ | 4 | Hamster sera |
|  |  |  |  |  | 0.8184 (IC50) | Linear R^2^ | 4 | Hamster sera |
|  |  |  |  |  | 0.6178 (IC50) | Linear R^2^ | 4 | Hamster sera |
| Neerukonda *et al*. 2021 (62) | SARS-CoV-2 | HIV | PRNT | Luciferase | 0.8066* (IC50) (p<0.001) | Spearman's | 14 | Human sera |
|  |  |  |  |  | 1* (IC50) (p=0.0028) | Spearman's | 5 | Human sera |
|  |  |  |  |  | 0.9197* (IC80) (p<0.0001) | Spearman's | 14 | Human sera |
|  |  |  |  |  | 1* (IC80) (p=0.0028) | Spearman's | 5 | Human sera |
| Newman *et al*. 2022 (63) | SARS-CoV-2 | HIV | CPE | luciferase | 0.70 (IC80) (p=0.0035) | Spearman's | 16 | Human sera |
| Nguyen *et al*. 2022 (64) | SARS-CoV-2 | HIV | ELISA | Luciferase | 0.9 (IC50) (p<0.001) | Linear R^2^ | 20 | Human plasma |
|  |  |  |  |  | 0.91 (IC50) (p<0.001) | Linear R^2^ | 20 | Human plasma |
|  |  |  |  |  | 0.91 (IC50) (p<0.001) | Linear R^2^ | 20 | Human plasma |
|  |  |  |  |  | 0.87 (IC50) (p<0.001) | Linear R^2^ | 55 | Human plasma |
|  |  |  |  |  | 0.78 (IC50) (p<0.001) | Linear R^2^ | 55 | Human plasma |
|  |  |  |  |  | 0.73 (IC50) (p<0.0001) | Linear R^2^ | 55 | Human plasma |
| Ni *et al*. 2020 (65) | SARS-CoV-2 | HIV |  | Luciferase | 0.9728 (IC90) (p<0.0001) | Linear R^2^ | 20 | Human sera |
| Noval *et al.* 2020 (66) | SARS-CoV-2 | HIV | Monolayer Score | Luciferase | 0.64 (IC50) (p<0.0001) | Linear R^2^ | 101 | Human sera |
|  |  |  |  |  | 0.4628 (IC90) (p<0.0001) | Linear R^2^ | 101 | Human sera |
| Oguntuyo *et al*. 2021 (67) | SARS-CoV-2 | VSV | NP-HRP | Luciferase | 0.8159 (IC50) (p<0.0001) | Pearson's | 15 | Human sera |
|  |  |  |  |  | 0.6415 (IC80) (p<0.0001) | Pearson's | 15 | Human sera |
| Schmidt *et al*. 2020 (68) | SARS-CoV-2 | VSV | Immunofluoresence | Luciferase | 0.73 (IC50) (p<0.001) | Pearson's | 20 | Human plasma |
|  |  |  |  |  | 0.86 (IC50) (p<0.0001) | Spearman's | 20 | Human plasma |
|  |  | HIV | Immunofluoresence | Luciferase | 0.82 (IC50) (p<0.0001) | Pearson's | 20 | Human plasma |
|  |  |  |  |  | 0.94 (IC50) (p<0.0001) | Spearman's | 20 | Human plasma |
|  |  | HIV | Immunofluoresence | Luciferase | 0.76 (IC50) (p<0.001) | Pearson's | 15 | mAbs |
|  |  |  |  |  | 0.91 (IC50) (p<0.0001) | Spearman's | 15 | mAbs |
|  |  | VSV | Immunofluoresence | Luciferase | 0.89 (IC50) (p<0.001) | Pearson's | 15 | mAbs |
|  |  |  |  |  | 0.92 (IC50) (p<0.0001) | Spearman's | 15 | mAbs |
| Sholukh *et al*. 2021 (41) | SARS-CoV-2 | HIV | Luciferase | Luciferase | 0.81 (IC50) (p<0.001) | Pearson's | 40 | Human plasma |
|  |  | VSV | Luciferase | Luciferase | 0.82 (IC50) (p<0.001) | Pearson's | 40 | Human plasma |
|  |  | HIV | Luciferase | Luciferase | 0.85 (IC50) (p<0.001) | Pearson's | 40 | Human plasma |
|  |  | HIV | Luciferase | Luciferase | 0.78 (IC50) (p<0.001) | Pearson's | 40 | Human plasma |
| Tan *et al*. 2020 (69) | SARS-CoV-2 | VSV |  | Luciferase | 0.7678 (IC50) (p<0.0001) | Linear R^2^ | 60 | Human sera |
| Von Rhein *et al.* 2021 (75) | SARS-CoV-2 | HIV | PRNT | Luciferase | 0.79 (IC50) | Linear R^2^ | 29 | Human plasma |
|  | SARS-CoV-2 | HIV | PRNT | Luciferase | 0.84 (IC50) | Linear R^2^ | 8 |  |
| Wang *et al*. 2020 (40) | SARS-CoV-2 | VSV | CPE | Luciferase | 0.83 (IC50) (p<0.0001) | Pearson's | 19 | Human sera |
|  |  |  |  |  | 0.82 (IC50) (p<0.0001) | Pearson's | 16 | Human sera |
| Wohlgemuth *et al*. 2021 (70) | SARS-CoV-2 | VSV | PRNT | SEAP | 0.771 (IC50) (p<0.0001) | Pearson's | 39 | Human sera |
|  |  |  |  |  | 0.757 (IC50) (p<0.0001) | Pearson's | 39 | Human sera |
|  |  |  |  |  | 0.703 (IC50) (p<0.0001) | Pearson's | 39 | Human sera |
| Xiong *et al*. 2020 (71) | SARS-CoV-2 | VSV | CPE | eGFP | 0.8396 (IC50) (p<0.0001) | Linear R^2^ | 12 | Human sera |
| Yang *et al*. 2020 (72) | SARS-CoV-2 | HIV | qPCR | Luciferase | 0.8325 (IC50) | Pearson's | 11 | Human sera |
|  |  |  |  |  | 0.6931 (IC50) (p<0.005) | Linear R^2^ | 11 | Human sera |
| Yu *et al*. 2021 (73) | SARS-CoV-2 | HIV | Luciferase | Luciferase | 0.747 (IC50) (p<0.0001) | Spearman's | 67 | Macaque sera |
| Zettl *et al*. 2020 (74) | SARS-CoV-2 | VSV | CPE | Luciferase | 0.939 (IC50) (p<0.001) | Pearson's | 13 | Human sera |
|  |  |  |  |  | 0.929 (IC50) (p<0.0001) | Pearson's | 25 | Human sera |
| Temperton *et al*. 2005 (76) | SARS-CoV-1 | MLV | CPE | GFP | 0.78 (IC50) | Pearson's | 11 | Human sera |
|  |  |  |  |  | 0.69 (IC90) | Pearson's | 11 | Human sera |
| Fukushi *et al*. 2006 (77) | SARS-CoV-1 | VSV | CPE | GFP | 0.77 (IC50) | Pearson's | 56 | Human sera |
| Logan *et al*. 2016 (78) | CDV | VSV | CPE | Luciferase | 0.76 (IC90) (p<0.0001) | Spearman's | 202 | Dog sera |
|  |  |  |  |  | 0.79 (IC90) (p<0.0001) | Spearman's | 202 | Dog sera |
|  |  |  |  |  | 0.65 (IC90) (p<0.0001) | Spearman's | 202 | Dog sera |
|  |  |  |  |  | 0.91 (IC90) (p<0.0001) | Spearman's | 168 | Dog sera |
| Kishishita *et al.* 2013 (79) | CHIKV | HIV | CPE | Luciferase | 0.98 (IC50) | Linear R^2^ | 4 | Mouse sera |
|  |  |  |  |  | 0.78 (IC50) | Linear R^2^ | 23 | Human sera |
| Wright *et al*. 2008 (80) | EBLV-1 | VSV | FAVN | Luciferase | 0.79* (IC100) | Pearson's | 9 | Animal Sera |
| Wright *et al*. 2008 (80) | EBLV-2 | VSV | FAVN | Luciferase | 0.9 (IC100) | Pearson's | 9 | Animal Sera |
| Konduru *et al*. 2018 (81) | EBOV | VSV | PRNT | FRNT | 0.96 (<0.0001) | Pearson's | 5 | Guinea pig sera |
|  |  |  | PRNT | PRNT | 0.96 (<0.0001) | Pearson's | 5 | Guinea pig sera |
| Steeds *et al*. 2020 (82) | EBOV | VSV |  | Luciferase | 0.86 (IC50) (p<0.0001) | Spearman's | 40 | Human plasma |
|  |  | VSV |  |  | 0.69* (IC50) (p<0.0001) | Spearman's | 30 | Human plasma |
|  |  | HIV |  |  | 0.54 (IC50) (p<0.001) | Spearman's | 40 | Human plasma |
|  |  | HIV |  |  | 0.38* (IC50) (p=0.0375) | Spearman’s | 30 | Human plasma |
| Wilkinson *et al*. 2017 (83) | EBOV | VSV |  |  | 0.99 | Unknown | 9 | Mixed serum and Abs |
|  |  | VSV |  |  | 0.84* | Unknown | 7 | Mixed serum and Abs |
|  |  | VSV |  |  | 0.99 | Unknown | 9 | Mixed serum and Abs |
|  |  | VSV |  |  | 0.96* | Unknown | 7 | Mixed serum and Abs |
|  |  | HIV |  |  | 0.68 | Unknown | 9 | Mixed serum and Abs |
|  |  | HIV |  |  | -0.03* | Unknown | 7 | Mixed serum and Abs |
|  |  | HIV |  |  | 0.77 | Unknown | 9 | Mixed serum and Abs |
|  |  | HIV |  |  | 0.18* | Unknown | 7 | Mixed serum and Abs |
| Wasilewski *et al*. 2016 (84) | HCV | HIV | ELIspot | Luciferase | 0.8 (IC50) (p=0.002) | Spearman's | 12 | mAbs |
|  |  |  |  |  | 0.7 (Single Dilution) (p<0.0001) | Spearman's | 34 | mAbs |
| Bailey *et al*. 2014 (85) | HCV | HIV | Fluorescence | Luciferase | 0.93 (IC50) (p<0.02) | Spearman's | 6 | mAbs |
| Urbanowicz *et al*. 2016 (86) | HCV | MLV | Immunofluoresence | GFP | 0.8938 (IC50) (p=0.0152) | Pearson's | 5 | mAbs |
| Chan et al. 2006 (87) | HIV | MMLV | Luciferase | beta-Gal | 0.903 (IC50) | Linear R^2^ | 70 | Antagonist Reagents |
| Li et al. 2017 (88) | HTNV | VSV | PRNT | Luciferase | 0.91 (IC50) | Linear R^2^ | 62 | Rabbit sera |
| Alberini *et al*. 2009 (90) | IAV H5N1 | HIV | ELISA | Luciferase | 0.78* (IC80) (p<0.001) | Pearson's | 226 | Human sera |
| Buchy *et al*. 2010 (91) | IAV H5N1 | HIV | CPE | Luciferase | 0.79 (IC50) (p<0.001) | Spearman's | 101 | Human sera |
| Garcia *et al*. 2009 (92) | IAV H5N1 | HIV | CPE | Luciferase | 0.734* (IC50) (p<0.001) | Pearson's | 26 | Human sera |
| Temperton *et al*. 2007 (93) | IAV H5N1 | MLV |  | GFP | 0.99 | Linear R^2^ | 5 | Human sera |
|  |  |  |  |  | 0.78* | Linear R^2^ | 56 | Human sera |
| Wang *et al*. 2010 (94) | IAV H5N1 | MLV | ELISA | β-Gal | 0.9802* (IC95) | Linear R^2^ | 17 | Mixed sera |
|  |  |  |  |  | 0.8193* (IC95) | Linear R^2^ | 17 | Mixed sera |
|  |  |  |  |  | 0.5244* (IC95) | Linear R^2^ | 17 | Mixed sera |
| Tian *et al*. 2018 (95) | IAV H7N9 | HIV | ELISA | Luciferase | 0.82 (IC50) (p<0.0001) | Pearson's | 339 | Human sera |
| Lee *et al*. 2022 (96) | JEV | JEV | PRNT | X-Gal | 0.9154 (IC50) (p<0.0001) | Linear R^2^ | 30 | Human sera |
| Wright *et al*. 2010 (97) | LBV | HIV | Luciferase | Luciferase | 0.83 (IC100) (p<0.0001) | Pearson's | 184 | Bat sera |
| Perera *et al*. 2013 (98) | MERS | HIV | CPE | luciferase | 0.88 (IC90) | Pearson's | 21 | Camel sera |
| Park *et al*. 2015 (99) | MERS | HIV | PRNT | Luciferase | 0.97 (IC90) | Spearman's | 95 | Human sera |
| Lester *et al*. 2019 (100) | MERS | VSV |  | Luciferase | 0.9348 (IC80) (p<0.0001) | Pearson's | 52 | Human sera |
| Alharbi *et al*. 2019 (101) | MERS | HIV | unclear | Luciferase | 0.96 (IC50) (p<0.0001) | Linear R^2^ | 8 | Camel sera |
| Wang *et al*. 2014 (102) | NDV | HIV | CPE | Luciferase | 0.92 | Linear R^2^ | 16 | Sera |
| Tamin *et al*. 2009 (103) | NIV | VSV | PRNT | Luciferase | 0.51 | Linear R^2^ | 363 | Pig sera |
|  |  |  |  |  | 0.62 | Linear R^2^ | 363 | Pig sera |
|  |  |  |  |  | 0.68 | Linear R^2^ | 363 | Pig sera |
| Logan *et al*. 2016 (104) | PPRV | VSV | Luciferase | Luciferase | 0.89 (IC90) (p<0.0001) | Spearman's | 72 | Mixed sera |
| Iheozor-Ejiofor *et al*. 2016 (105) | PUUV | VSV | FRNT | GFP | 0.82* (IC50) | Spearman's | 17 | Human sera |
| Bukbuk *et al*. 2014 (106) | RFV | VSV | CPE | Luciferase | 0.77 (IC50) | Spearman's | 278 | Human sera |
| Nie *et al*. 2017 (107) | RABV | HIV | RFFIT | luciferase | 0.946 (IU/mL) | Linear R^2^ | 295 | Human and Mouse sera |
| Wright *et al*. 2009 (108) | RABV | HIV | FAVN | Luciferase | 0.915 (IC50) | Pearson's | 304 | Dog sera |
|  | RABV | HIV | FAVN | β-Gal | 0.918 (IC50) | Pearson's | 33 | Dog sera |
| Ning *et al*. 2021 (89) | SEOV | VSV | PRNT | Luciferase | 0.82 (IC50) | Linear R^2^ | 62 | Rabbit sera |
| Li *et al*. 2017 (88) | SEOV/HTNV | VSV | PRNT | Luciferase | 0.845 (IC50) (p<0.001) | Linear R^2^ | 44 | Human sera |
